# Aggressive infantile myofibromatosis with intestinal involvement

**DOI:** 10.1186/s40348-021-00117-9

**Published:** 2021-06-16

**Authors:** Tristan Römer, Norbert Wagner, Till Braunschweig, Robert Meyer, Miriam Elbracht, Udo Kontny, Olga Moser

**Affiliations:** 1 Division of Pediatric Hematology, Oncology and Stem Cell Transplantation, Medical Faculty, RWTH Aachen University, Pauwelstrasse 30, 52074 Aachen, Germany; 2 Institute of Pathology, RWTH Aachen University, Aachen, Germany; 3 Institute of Human Genetics, RWTH Aachen University, Aachen, Germany

**Keywords:** Infantile myofibromatosis, *PDGFRB* mutation, Intestinal polyposis, Molecular targeted therapy

## Abstract

**Background:**

Infantile myofibromatosis (IM) is the most common cause of multiple fibrous tumors in infancy. Multicentric disease can be associated with life-threatening visceral lesions. Germline gain-of-function mutations in *PDGFRB* have been identified as the most common molecular defect in familial IM.

**Case presentation:**

We here describe an infant with *PDGFRB*-driven IM with multiple tumors at different sites, including intestinal polyposis with hematochezia, necessitating temporary chemotherapy.

**Conclusions:**

*PDGFRB*-driven IM is clinically challenging due to its fluctuating course and multiple organ involvement in the first years of life. Early molecular genetic analysis is necessary to consider tyrosine kinase inhibitor treatment in case of aggressive visceral lesions.

## Background

Infantile myofibromatosis (IM) is a rare neoplastic condition with a reported incidence of 1 in 150,000 live births, but one of the most common causes of fibrous tumors in infancy [1]. It is characterized by the development of single (solitary IM) or multiple nodular masses in the soft tissues, bones, or seldom viscera (multicentric IM with or without visceral involvement) [2]. A detailed assessment including imaging procedures is necessary to document number of lesions, size of each lesion, and proximity to vital organs [3]. Diagnosis of IM can be challenging, but is usually confirmed by characteristic histologic and immunohistochemical findings [4]. Despite its general benign course with spontaneous regression during childhood, the multicentric form of IM can be life-threatening due to visceral involvement, which most often affects the cardiopulmonary and gastrointestinal system. These cases are associated with high mortality, and treatment is challenging, frequently necessitating systemic chemotherapy to prevent progressive organ damage [1, 5–7].

Activating somatic and germline point mutations in the *PDGFRB* gene, encoding for the PDGFRb tyrosine kinase, which is mainly expressed in cells of mesenchymal origin, are a frequent cause of sporadic or familial IM [8–10]. Activation of the PDGFRb tyrosine kinase receptor drives multiple important intracellular signal pathways, including the Ras/MAPK pathway, thus promoting cell growth and survival [11]. The mutation c.1681C>T is the most frequent *PDGFRB* mutation in IM and has been shown to constitutively activate the PDGFRb receptor, eventually leading to cancer development [10–12].

We here describe a familial case of infantile myofibromatosis, harboring the characteristic *PDGFRB* mutation c.1681C>T, with a remarkable clinical course in terms of spatial and temporal distribution of lesions, including intestinal manifestation with indication for chemotherapy. This case illustrates the challenges the clinician can be faced within the management of multicentric IM.

## Case presentation

**Fig. 1:**
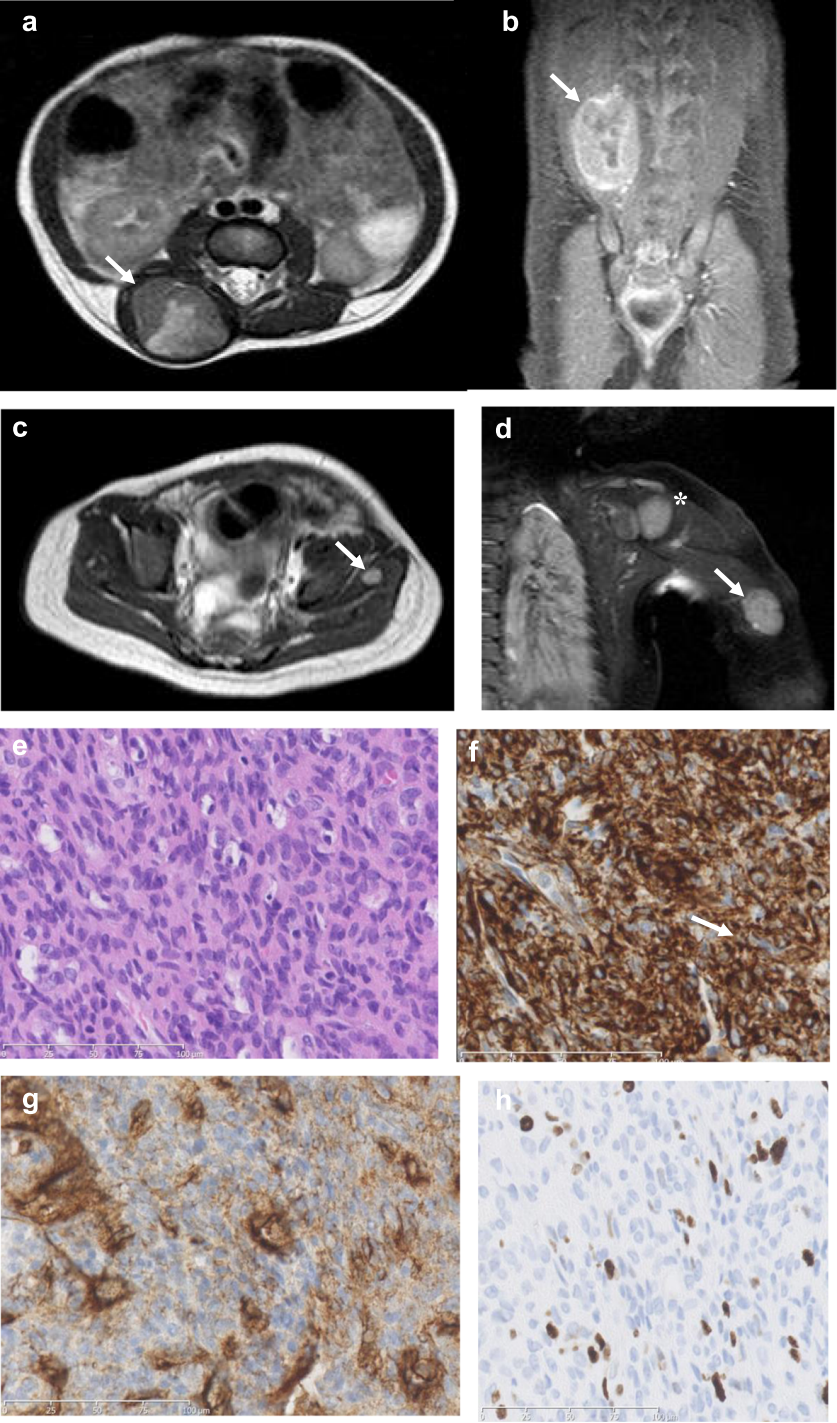
Imaging of the initial paraspinal tumor and additional intramuscular lesions and histopathology of the index tumor. MRI demonstrated a T2-inhomogeneous tumor within the paraspinal musculature (**a**), showing central necrosis and strong contrast enhancement (coronal T1-weighted imaging in **b**). Additional tumor foci in the left psoas muscle (**c**) and left triceps muscle (**d**), each smaller than the index lesion, but with similar T2 signal intensity (asterisk in d indicates humeral head). Histopathology of the paraspinal tumor showed dense spindle-shaped tumor cells and a prominent intratumoral capillary network (hemangiopericytoma-like pattern) (hematoxylin-eosin stain, × 400, in **e**). In immunohistochemical analysis, tumor cells stained strongly positive for WT1 (**f**), but largely negative for SMA and desmin (not shown), while intratumoral vessels stained positive for CD34 (**g**). Tumor cells displayed only low Ki-67 labeling index of 1–2% (**h**)

Our patient is a female child that was born full-term after an uneventful pregnancy. Immediately after birth, a firm non-tender soft tissue mass beside the lumbar spine was noted. Sonographic assessment showed a well-circumscribed, vascularized soft tissue mass, measuring 3.6 × 2.8 × 1.7 cm, localized in the right paraspinal muscles. MRI at the age of 10 days demonstrated inhomogeneous T2 hyperintensity and contrast enhancement of the lesion (Fig. 1a, b), but also revealed multiple additional tumor foci in the muscles of all four extremities, each smaller than the index lesion, but with the same imaging characteristics (Fig. 1c, d). Due to malignant appearance on MRI, biopsy of the paraspinal index lesion was performed the next day. Histopathology showed spindle-shaped cell infiltration with prominent capillary vasculature and small necrotic areas, surrounded by a hyaline matrix (Fig. 1e). On immunohistochemical analysis, tumor cells stained strongly positive for WT1 (Wilms’ tumor transcription factor-1) (Fig. 1f), but largely negative for SMA and desmin. Extensive tumor vascularization was indicated by immunopositivity for CD34 (Fig. 1g), a well-established marker for diverse progenitor cells, including vascular endothelial progenitors [13]. The Ki67 index was only 1–2%, indicating low proliferative activity (Fig. 1h). Clinical and histological picture was consistent with the diagnosis of infantile myofibromatosis with hemangiopericytoma-like pattern. Because of the general benign course of such lesions, a watch-and-wait strategy with MRI every three months was initially favorized.

**Fig. 2:**
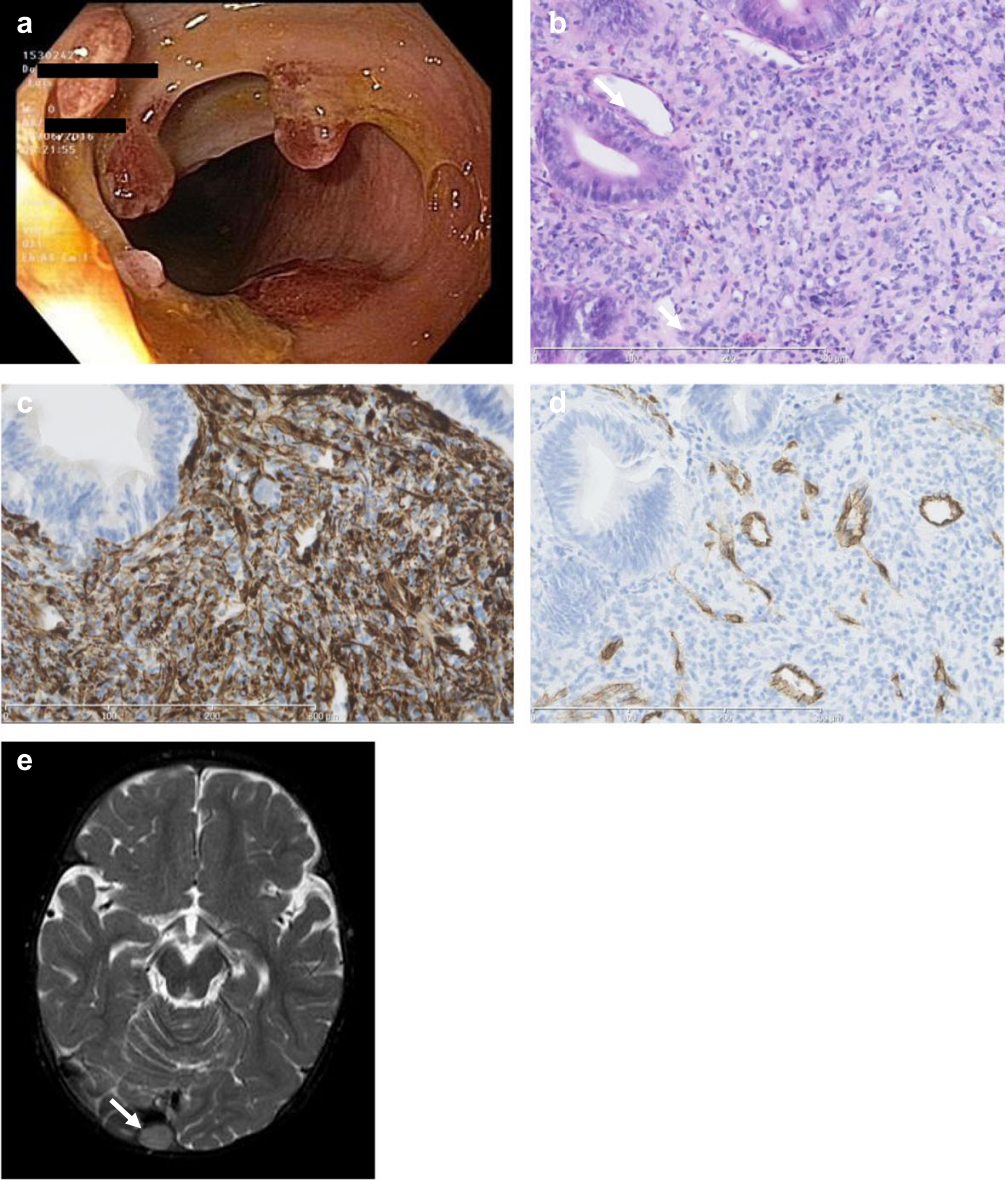
Further tumor lesions during the disease course. Colonoscopy revealed multiple polyps in the descending colon (**a**). Histopathology and immunohistochemistry of intestinal polyposis revealed a strikingly similar histomorphological picture (hematoxylin-eosin stain, × 20, in **b**) and immunoreactivity pattern for WT1 (**c**) and CD34 (**d**) as compared to the paraspinal index tumor in Fig. 1. MRI of the brain, showing a T2-hyperintense tumor lesion originating from the occipital skull with close proximity to the confluens sinuum (**e**)

During the following 6 months, the intramuscular lesions spontaneously regressed. However, at the age of 5 months, the child developed intermittent hematochezia with consecutive anemia (hemoglobin 79 g/l). Cow’s milk protein allergy was initially suspected, but elimination diet of the nursing mother did not lead to cessation of rectal bleedings. Colonoscopy was performed at the age of 6 months, and at least fifteen polyps were detected in the transverse, descending, and sigmoid colon, with sessile morphology and a maximum size of 1.5 cm (Fig. 2a). As no active bleeding source could be identified on endoscopy, polypectomy was not undertaken, but instead, only biopsy of three polyps was done. Histopathology and immunohistochemical pattern showed striking similarity to the initial paraspinal tumor (Fig. 2b–d).

Because of symptomatic polyposis with intermittent hematochezia and consecutive anemia, we decided to treat the child with a chemotherapeutic regimen of vincristine, actinomycin-D, and cyclophosphamide (VAC), according to the CWS guidance for non-resectable aggressive fibromatosis [14]. Each VAC course consisted of vincristine 1.5 mg/m^2^ once weekly over 3 weeks and actinomycin-D 1.5 mg/m^2^ and cyclophosphamide 20 mg/kg each as a single dose on day 1.

After initiation of chemotherapy, no further episodes of hematochezia could be noted. Endoscopic reassessment after three VAC courses showed a decrease in size of polyps, so we decided to continue chemotherapy. After six VAC courses, we also noted a significantly decreasing number of polyps, as well as complete remission of the intramuscular lesions, so we then decided to omit further chemotherapy. Follow-up management was supplemented by endoscopic assessments every 3 months for early detection of progressive polyposis.

**Fig. 3:**
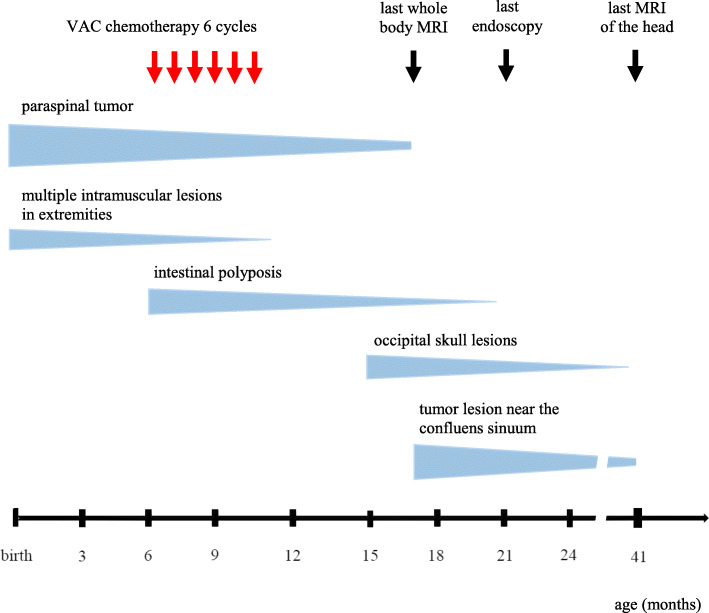
Timeline of detection and subsequent regression of different tumor manifestations in our patient. Thickness of bars qualitatively represents size of tumor lesions, with the paraspinal index tumor being the largest one (Ø 3 cm). After intestinal polyposis was detected, VAC chemotherapy was applied. All other manifestations were asymptomatic and thus managed by a watch-and-wait strategy. All tumor lesions showed continuous regression throughout the second year of life, as shown by decreasing thickness of bars. Please note that last whole body MRI at the age of 17 months revealed a small residual mass of the initial paraspinal tumor, and last MRI of the head at the age of 41 months also showed residual changes near the confluens sinuum, so these two lesions had not completely disappeared to the date of last MRI

Further disease course in our patient was characterized by a fluctuating picture. While intestinal polyposis was endoscopically undetectable 1 year after omission of chemotherapy, two new superficial lesions in the occipital region with a maximum size of 1 cm had emerged at the age of 15 months. These lesions were located to the tabula interna of the skull bone by means of MRI, clearly delineated from the intracranial space by the cortical bone. Two months later, while these two lesions already showed regression in size, another lesion in close proximity to the confluens sinuum, presumably also originating from the skull bone, was newly detected (Fig. 2e). Due to lack of symptomatic brain compression or sinus vein obstruction, we decided to not immediately apply any local or further systemic treatment, but instead, a close watch-and-wait strategy was pursued, with close clinical controls and MRI every 3 months, for detection of progressive tumor growth and brain compression. Fortunately, further clinical course was uneventful, and all lesions continuously regressed throughout the second year of life. Since her second birthday, no further lesions have evolved, and to the age of 5 years at the time of this report, the child has been in good health. All aforementioned lesions have further regressed, with last MRI of the head at the age of 3 years. The timeline of detection and subsequent regression of tumor manifestations in our patient is illustrated in Fig. 3.

Family history revealed that both the child’s father and one half-brother from the father’s side had spontaneously remitting subcutaneous nodules in infancy, with the half-brother being histologically diagnosed with “cutaneous leiomyoma.” In context of the positive family history and to explore the possibility of targeted therapy in case of relapse, germline genetic analysis was initiated. The characteristic missense mutation c.1681C>T in exon 12 of the *PDGFRB* gene (transcript ID NM_002609.3) was found in DNA of patient’s lymphocytes. Segregation analysis identified the mutation in the child’s father and her half-brother as well.

## Discussion and conclusions

This case of infantile myofibromatosis showed a remarkable disease course in terms of spatial and temporal distribution of lesions, necessitating chemotherapy due to symptomatic visceral involvement. Critical lesions in our patient were located in the gastrointestinal tract leading to hematochezia and the skull with proximity to the confluens sinuum.

There are several reports of involvement of the gastrointestinal tract in IM in the literature. Its clinical manifestations most often include chronic diarrhea, poor feeding, and weight loss, but also can comprise acute life-threatening events such as bowel obstruction, perforation, or intussusception [15–18]. Ongoing hematochezia with development of anemia, as we observed in our patient, has not been specifically mentioned as a leading symptom before in the literature. Early endoscopic assessment and treatment is warranted in this scenario to prevent further complications. Intestinal polyposis with bleeding led us to treat the child with chemotherapy, inducing a rapid clinical response with cessation of hematochezia. After six VAC courses, a marked decrease in the number of intestinal lesions could be noted on endoscopy. We considered this to reflect a chemotherapy effect, but regression of the intestinal lesions further continued after omission of chemotherapy. How chemotherapy contributed to remission of IM-associated intestinal polyposis in our case therefore remains speculative. However, due to relevant bleeding and the potential for life-threatening events, as described in cases with intestinal involvement, prompt intervention was deemed necessary.

CNS involvement is even much rarer in IM, but can lead to brain or spinal compression [19–21]. Our patient developed a tumor lesion in close proximity to the confluens sinuum. The anatomical origin of this lesion could not be clearly determined by means of MRI, but it was assumed to be rather of osseous than dural origin, as two other lesions in this region were clearly located to the tabula interna of the skull bone. However, as this lesion showed no relevant compression of the brain and could be clearly demarcated from the sinus vein system, we decided to not immediately apply any further treatment in an asymptomatic child by weighing out risks and benefit, considering potential complications of surgery and cumulative toxicities with further chemotherapy. We instead employed a watch-and-wait strategy with close MRI controls to early detect progressive tumor growth and brain compression. Fortunately, the lesion showed spontaneous regression during further assessments, and no local or further systemic treatment was necessary. However, our management surely is debatable, considering the unpredictable growth dynamics of this critically located lesion. All in all, the clinical course in our patient impressively demonstrates how tumors in *PDGFRB*-driven IM arise temporally separated during the first years of life, with each lesion showing individual growth characteristics. Growth and regression of tumors can occur simultaneously in various tissues during this vulnerable period. However, tumor development and growth seems not to be strictly restricted to early childhood, as there are reports of adolescents and young adults with *PDGFRB*-driven IM, in whom tumor manifestations recurred or first occurred at a later age [21–23]. Al Qawahmed et al. reported intracranial tumor growth in an adolescent girl with *PDGFRB*-driven IM [21]. Another report described a female with recurrence of tumor manifestations at the age of 24 years during pregnancy [22]. These reports suggest that hormonal influences during puberty or pregnancy might play a role in the initiation of tumor growth on the background of an activating *PDGFRB* germline mutation. However, molecular mechanisms triggering tissue- and age-specific tumor growth, regression, or recurrence in *PDGFRB*-driven IM largely remain obscure to date. At the time when diagnostic and treatment decisions had to be made in our patient, no specific guidelines existed to support decision-making, so management was mainly based on the individual clinical course. Recently, expert recommendations for the diagnosis and surveillance of patients with IM were published [24]. As all tumor manifestations in our patient showed regression throughout the second year of life, and no further symptomatic lesions evolved after the child’s second birthday, we discontinued regular sonographic and MRI assessments thereafter. Last whole body MRI at the age of 17 months and last MRI of the head at the age of 3 years demonstrated residual changes of the paraspinal index tumor and the cranial lesion near the confluens sinuum, while all other subcutaneous, muscular, and bone lesions had completely regressed. However, after the period of highest mortality in early childhood and subsequent tumor regression in most patients, it is important not to lose these patients and their families during follow-up, as there is a life-long risk for tumor recurrences and cerebral aneurysms in individuals with *PDGFRB* germline mutations [24].

Activating *PDGFRB* mutations have been identified in most patients with multicentric IM and mainly seem to affect children. In one study, *PDGFRB* mutations were found in 9/15 children less than 2 years of age with multicentric IM, but not in adults with solitary myofibroma. It has therefore been proposed that *PDGFRB*-driven multicentric IM should be seen as a distinct entity of soft-tissue neoplasms in childhood [25]. We found the characteristic point mutation c.1681C>T in exon 12 of the *PDGFRB* gene, leading to the substitution of arginine to cysteine at codon 561 (p.Arg561Cys). This mutation has been found recurrently in cases of familial IM. In fact, 17 out of 21 affected families, in whom a heterozygous *PDGFRB* germline variant was found, were reported to carry the p.Arg561Cys variant. Clinical information is available for 19 individuals with multicentric disease from these 17 families [9, 18, 21–23, 26, 27]. To compare our case with the published ones and possibly find a recurrent phenotype, we reviewed clinical data on published cases of multicentric IM, in which the p.Arg561Cys germline variant of the *PDGFRB* gene was detected (Table 1). Regarding morbidity during early childhood, extent of involved anatomic sides, and frequent regression of tumor lesions by the age of 4 years at latest, our case fits well to the ones described in the literature. However, a wide clinical spectrum ranging from asymptomatic soft tissue nodules to multiple visceral manifestations resulting in life-threatening complications can be noted, even among individuals from one family. Of particular note is the case of a neonate described by Ortiz et al., who suffered from multiple organ involvement including severe gastrointestinal complications with need for bowel resection, and who died due to cardiorespiratory failure, showing that multicentric IM is a potentially lethal condition [18]. On the other hand, many patients only develop multiple nodules of the skin or subcutaneous tissue with spontaneous regression during childhood and no further organ involvement [9]. The head-neck region seems to be a frequently involved anatomic location, and bone involvement is common [9, 18, 22, 23, 26]. Most cases are diagnosed immediately after birth or during early childhood. However, the cases described by Murray et al. and Weller et al. show that tumor development due to the *PDGFRB* mutation p.Arg561Cys is not restricted to childhood, but requires life-long awareness of an increased tumor risk [22, 23]. It needs to be emphasized that most reports lack detailed information on individual clinical outcomes, hampering long-term prognostication in this rare and phenotypically heterogeneous disease.

**Table 1:** Clinical data on published cases of multicentric IM due to confirmed p.Arg561Cys germline mutation in the *PDGFRB* gene

Reference	Cases	Age at first presentation	Clinical manifestations	Outcome
[9]	10 individuals from 4 families	3 weeks–4 years (indicated for 6 patients)	Multiple skin and subcutaneous myofibromas in all, orbital and supranasal mass in one patient	Spontaneous regression indicated for 5 patients, including 2 with documented remission at age 4 years, surgical excision in one patient, no further information
[18]	1 male infant	Birth	Skin, eye, bone, brain, heart, lung, and gastrointestinal involvement, intestinal obstruction and perforation, failure to thrive	Bowel resection, treatment with vinblastine and methotrexate, died in infancy due to cardiorespiratory failure
[21]	1 female adolescent	Infancy (< 24 months)	Multiple nodules in infancy, extradural tumor in the right posterior fossa at age 14 years	Spontaneous regression of manifestations in infancy, surgical excision of intracranial tumor
[22]	2 individuals from 1 family (mother and daughter)	Birth (both)	Mother: rectal bleeding after birth (no cause identified on endoscopy), multiple subcutaneous nodules in 1st year of life, spinal bone lesions, pancreas tumor at age 18 months Daughter: multiple subcutaneous nodules, intracranial mass originating from temporal bone at age 10 months	Mother: spontaneous regression at age 3 years, recurrence of subcutaneous tumors at age 24 years during pregnancy Daughter: spontaneous reduction of intracranial mass after 3 months, no information on further course
[23]	1 male adult	19 years (age at diagnosis 34 years)	Multiple recurring cutaneous, pulmonary, cranial, intraspinal, and muscular paraspinal lesions	Sustained regression of most lesions 12 months after initiation of treatment with imatinib, multiple previous treatments (chemotherapy, surgery, stereotactic radiotherapy)
[26]	2 siblings	♀: 5 months, ♂: birth	♀: two congenital nodules ♂: multiple nodules and toe necrosis at birth, cranial tumor, and bone lesion in toe at age 11 months	♀: surgical excision, no relapse to age 6 years ♂: no information on further course
[27]	2 individuals from 1 family	Not indicated	Severe refractory multicentric IM in index patient, no further information on clinical manifestations, congenital splenic tumor in the other patient	Treatment of index patient with vinblastine and methotrexate, no information on further course

Note that cases of solitary IM (2 patients) and asymptomatic mutation carriers (2 patients) are not included. Mudry et al. described a family with 2 individuals affected by multicentric IM due to the mutation p.Arg561Ser, who are also not included in this list [28]

The p.Arg561Cys mutation presumably disrupts the inhibitory juxtamembrane domain of PDGFRb, leading to constitutive activation of its kinase domain. The Arg561 residue seems to have a key function for the juxtamembrane-kinase domain interaction and is the most frequently mutated residue in IM families [9, 29]. *PDGFRB* mutations show incomplete penetrance and variable expressivity, indicating additional genetic modifiers. These probably contribute to the marked phenotypic heterogeneity even within affected families, as noted in our case and others. Among the 17 reported families with the p.Arg561Cys mutation, two mutation carriers had solitary IM and another two carriers were completely asymptomatic [9, 18, 22, 26]. Indeed, the p.Arg561Cys mutation has been shown to only weakly activate the PDGFRb receptor in fibroblasts; hence, it has been proposed that a second hit is necessary to fully activate the receptor, leading to tumor development and probably influencing extent and aggressiveness of tumor lesions [8]. Linhares et al. reported a family in whom two siblings had multicentric IM due to the p.Arg561Cys mutation, but the mother was asymptomatic although carrying the same mutation. In both affected siblings, a second heterozygous mutation in the *PTPRG* gene was found, eventually leading to impaired dephosphorylation of the already hyperactivated PDGFRb receptor, further promoting intracellular signal transduction and inducing excessive cell proliferation [26]. Investigating all cases with a confirmed p.Arg561Cys mutation for additional genetic lesions modifying the PDGFRb signaling pathway would show if the *PDGFRB* mutation alone is sufficient to cause severe IM or if second hits are essential for emergence of the full disease phenotype. This would allow a more precise molecular classification and prognostication. To date, the unpredictable disease course of *PDGFRB*-driven IM due to the lack of a defined genotype-phenotype correlation poses a challenge for the clinician who always must weigh out benefits and risks of aggressive treatment. Frequent assessment of lesions by repeated imaging studies is necessary to support decision-making, which always has to involve clinical characteristics, anatomic side, and growth dynamics of each lesion.

The p.Arg561Cys mutation has shown sensitivity to different kinase inhibitors, such as imatinib and sunitinib, offering a targeted therapy approach in affected patients [12, 23, 25, 28, 30, 31]. At the time of symptomatic visceral manifestation in our patient, the underlying genetic mutation in *PDGFRB* was unknown, and data about TKI treatment were scarce. We therefore applied VAC chemotherapy, according to the CWS treatment guidance for non-resectable aggressive fibromatosis. The VAC chemotherapy combination has yielded good responses in the treatment of aggressive fibromatosis, including 6 cases of multicentric IM, in the CWS-96 study, but cumulative toxicities and late sequelae are a concern in the young child [32]. Less intensive regimens such as vincristine and dactinomycin without cyclophosphamide have shown similar efficacy, but with less acute and long-term toxicity [6]. Another frequently applied chemotherapy regimen is the combination of vinblastine and methotrexate [18, 27, 28, 33]. However, due to the paucity of data regarding efficacy and safety of conventional chemotherapy in IM, its use should be restricted to cases with vital endangerment. Although VAC chemotherapy was well tolerated in our patient, TKI treatment would have also been a reasonable option, given the potential for severe toxicities with conventional chemotherapy and the recent reports about the successful use of TKI in *PDGFRB*-driven IM [23, 28, 31]. However, side effects, such as growth deceleration, are also a concern in TKI treatment, as has been described in children with chronic myeloid leukemia [34]. Treatment with imatinib is often accompanied by gastrointestinal side effects, fatigue, muscle cramps, and renal impairment, sometimes necessitating dose reductions or treatment interruptions [23]. Furthermore, the time to clinical response to imatinib in *PDGFRB*-driven IM is not yet defined, and its immediate efficacy in cases with vital endangerment due to critical tumor growth is doubtable. Mudry et al. reported a child with multicentric IM due to p.Arg561Ser germline mutation in *PDGFRB* who showed a rapid clinical response to treatment with sunitinib after 4 weeks, but developed grade 3–4 neutropenia and one episode of severe hypoglycemia [28]. It is assumed that targeted therapy against PDGFRb as a more selective approach compares favorably to the toxicity of conventional chemotherapy, but the full spectrum of adverse effects and long-term sequelae is still not known. If TKI monotherapy is sufficient to induce and sustain full disease remission is also a matter of debate, as most reports describe combination with conventional chemotherapy or second-line use in heavily pretreated patients [23, 28]. Furthermore, the necessary duration of TKI treatment in a disease caused by a germline mutation that confers a life-long tumor risk, but shows incomplete penetrance with a highly variable clinical course, remains elusive. Therefore, PDGFRb-targeted therapy offers the opportunity for a highly personalized treatment with acceptable side effects, but many issues regarding its optimal use in IM remain unsolved to date.

In conclusion, we here describe a case of familial *PDGFRB*-driven IM with special emphasis on the clinical challenges associated with an aggressive and fluctuating disease course. We especially highlight the unusual visceral manifestation with intestinal polyposis, hematochezia, and consecutive anemia. The endangerment of vital structures in the first years of life due to aggressive expansion and critical location of tumor lesions is in contrast to the further course with regression of apparently all lesions throughout childhood. The challenge is not to miss out progressive organ deterioration and need for treatment in this critical time period, but also not to lose patients thereafter, as there is ongoing risk for tumor development and recurrences. A thorough genetic investigation should early be initiated in cases of multiple fibrous tumors in infancy, as this could (1) be of diagnostic value in a histologically challenging disease and (2) offer the opportunity for molecular targeted therapy in patients with aggressive tumor expansion. Therefore, genetic counseling is nowadays of utmost importance in diagnosis, prognosis, and therapy guidance in IM. However, more data are needed to define genotype-phenotype correlations and thus help in clinical decision-making and prognostication for patients with IM tumor predisposition.

## Data Availability

Data sharing is not applicable to this article as no datasets were generated or analyzed.

## Abbreviations

CNS: Central nervous system
CWS: *Cooperative Weichteilsarkom* study group
IM: Infantile myofibromatosis
MRI: Magnetic resonance imaging
PDGFRB: Platelet-derived growth factor receptor-beta
PTPRG: Protein tyrosine phosphatase receptor-gamma
SMA: Smooth muscle actin
TKI: Tyrosine kinase inhibitor
VAC: Vincristine, actinomycin-D, cyclophosphamide
WT1: Wilms’ tumor transcription factor-1
